# Undetectable Anti-HBs Antibodies: Need of a Booster Dose for HIV-1-Infected Individuals

**DOI:** 10.3390/vaccines9121484

**Published:** 2021-12-15

**Authors:** Yonas Bekele, Jay A. Berzofsky, Francesca Chiodi

**Affiliations:** 1 Vaccine Branch, Center for Cancer Research, National Cancer Institute, National Institutes of Health, Bethesda, MD 20892, USA; berzofsj@mail.nih.gov; 2 Department of Microbiology, Tumor and Cell Biology, Biomedicum, Karolinska Institutet, Solnavägen 9, 17165 Solna, Sweden; francesca.chiodi@ki.se

**Keywords:** HBV, HBV vaccine, anti-HBs antibodies, HIV-1, booster dose, B cells, Tfh cells, HIV-HBV co-infection

## Abstract

HBV vaccination effectively prevents HBV transmission and the development of liver cancer. Disease progression and liver-related complications are more common in HIV-1/HBV co-infected than HBV mono-infected individuals. A considerable body of literature, which will be reviewed here, indicates that response to HBV vaccine is suboptimal in HIV-1-infected individuals and that the poor maintenance of protective immunity to HBV vaccines in these individuals is an important medical issue. Several factors affect HBV vaccine response during HIV-1 infection including CD4+ T cell counts, B cell response, vaccine formulation, schedules, and timing of antiretroviral therapy (ART). The initial response to HBV vaccination also plays a critical role in the sustainability of antibody responses in both HIV-1-infected and uninfected vaccinees. Thus, regular follow-up for antibody titer and a booster dose is warranted to prevent HBV transmission in HIV-1 infected people.

## 1. Introduction

Vaccines are among the most powerful health interventions and have significantly contributed to prolonging life expectancy worldwide. In this regard, hepatitis B virus (HBV) vaccination is the ideal example for the prevention of chronic infection and related complications including liver cirrhosis and hepatocellular carcinoma (HCC). An HBV vaccine became commercially available in 1982, initially as HBsAg (HBV surface antigen) isolated from patients, and later as a recombinant protein [1], and has proven a 95% efficacy in preventing HBV infection and associated symptoms [2]. Globally, the incidence of HBV infection and death due to chronic HBV infection-related complications declined over the years due to effective nationwide early childhood vaccination [3]. Still, in 2019 alone, 1.5 million and 820,000 people were newly infected and died of HBV-related complications, respectively, according to a WHO report [4]. Some of the challenges of HBV vaccination are that a proportion, corresponding to approximately 10% of vaccinated individuals, do not respond to the vaccine and that a decline of vaccine-specific antibodies to HBV surface (HBs) protein takes place in vaccinated individuals [5].

In a comprehensive study gathering data from individuals born in Israel after the introduction of the HBV vaccine and subsequently tested for anti-HBs Abs, a decline over time in levels of anti-HBs Abs was observed with 66.7% of adolescents scoring negative for HBV antibodies 15 years after vaccination [6]. The mechanism(s) leading to waning humoral responses to the HBV vaccine are not fully understood; it has been proposed that initial lack of response to the vaccine may be caused by specific HLA alleles or lack of cytokine production (reviewed in [7]) and overlapping mechanisms may likely account for the waning of immune responses. Interestingly, Klinger and colleagues [6] also showed that a booster dose of HBV vaccine provided to HBV seronegative subjects, previously receiving HBV vaccine, led to seroconversion in 93.8% of individuals; this study clearly showed that an anamnestic response to HBV vaccine is present in immunocompetent individuals despite a seronegative status.

In contrast, the waning of HBV vaccine responses is a major health problem connected to HIV-1 infection. A poor serological response to primary HBV vaccination and a rapid decline of anti-HBs antibody levels, already within the first year from vaccination, has been reported in HIV-1 infected individuals [8,9,10,11,12]; the gradual decline of protective immunity to the HBV vaccine becomes more noticeable over time in HIV-1 infected individuals. As extensively reviewed, the burden of HIV-1 is higher in Sub-Saharan Africa, though a significant decrease in mortality and new cases in 2019 compared to 1990 was reported [13]. Sub-Saharan Africa and East Asia are considered to be high chronic HBV endemic regions and around 10% of HIV-positive individuals are co-infected with HBV [14].

This is an important medical concern considering that people with co-morbidity and/or immunocompromised individuals especially with HIV have a higher chance of developing chronic HBV infection and may also die with liver-related complications. The present review aims at summarizing current knowledge on responses to HBV vaccination and the need for a booster dose in HIV-1-infected individuals. (Methodology: To retrieve relevant literature in Pubmed and Web of Science we used the following keywords: Hepatitis B virus”, “HBV”, “HBV burden”, “chronic HBV”, “HIV”, “HIV-HBV coinfection”, “Vaccine response”, “anti-HBs”, “B cells”, and “Tfh cells”. All articles with full text were included, and those lacking vaccine dosage, terms of protection, schedules and rate of response were excluded.)

## 2. HBV Infection in HIV-1 Infected Individuals

HBV infection is generally self-limiting in immunocompetent adults; the likelihood of clearing HBV infection is, however, lower in HIV-1-infected compared to healthy individuals. Acute HBV infection can cause acute liver failure and death in both HIV-1-infected and uninfected individuals [15]. The pathogenic effects of HBV and HIV-1 infections are considerably worsened in co-infected patients [16,17]. Elevated levels of HBV DNA, reduced ability to clear circulating hepatitis B envelope antigen (HBeAg), and a higher risk of developing chronic HBV infection were reported in co-infected, compared to HIV-1-negative individuals [16,18]. In addition, the progression rate to cirrhosis and/or HCC is much faster, and liver-related mortality is more common in co-infected individuals [16,18].

Besides, break-through HBV infections were also identified in fully vaccinated HIV-1-infected children [19], in HIV-1 infected adolescents [20], and even in HIV-1 uninfected individuals [21,22,23]. In some of these acutely infected individuals, the infection resolved with negative results upon subsequent testing for HBV DNA and HBeAg [20,23]. In some previous reports, the source of HBV infection in vaccinated HIV-1-infected children was not regularly identified, but the virus could be transmitted from HBsAg positive mothers [19,22], occult HBV infection [23], or exposure to vaccine-escape mutant HBV strains; in the latter context, vaccine-induced protective antibodies are ineffective to neutralize some HBV S gene mutants [24].

Anti-retroviral therapy (ART) effectively slows down disease progression and reduces the death rate in the HIV-1/HBV co-infected individuals [25]. However, liver toxicity is a common side effect of ART in HIV-1/HBV-infected individuals [25]. Besides, drug resistance to lamivudine, which is the most effective drug for the treatment of HBV infection, and cross-drug resistance to other anti-HBV drugs, hampered the global effort to slow down disease progression in co-infected people [25]. Other compounds including interferon-α, pegylated interferon-α, adefovir, entecavir, telbivudine, and tenofovir are used to reduce liver damage and to prevent long-term complications of HBV infection in mono-infected or HBV-HIV-1 co-infected patients [26]. Thus, patients with HIV/HBV still need the best, effective, affordable, and accessible treatment to prevent liver-related complications and death.

## 3. The Impact of Immunological and Virological Parameters on Response to HBV Vaccine in HIV-1 Infected Individuals

The most common schedule for HBV vaccination is three doses of the vaccine at 0–1–6 months; however, an accelerated vaccination schedule at 0–4–8 weeks is also recommended for high-risk individuals to increase vaccine compliance and elicit higher antibody responses. In the latter schedule, a fourth dose was recommended to slow down the decline of anti-HBs antibodies and for long-term protection. Several studies in different settings showed a decline in the anti-HBs antibodies a few or several years after vaccination, depending on the vaccination strategies and other factors. Lower response to HBV vaccine and short duration of protection were reported in HIV-1-infected individuals. Surprisingly, a suboptimal HBV vaccine response was reported even in HIV-exposed but uninfected children compared to HIV unexposed children [27]. Several factors could be implicated in impaired immune responses to HBV vaccine in HIV-1-infected individuals and the risk of acquiring chronic HBV infection, including low CD4^+^ T cell count, viral load, time of ART initiation, vaccination schedule, vaccine formulations, gender, and co-morbidities, as elaborated below.

In support of the role of CD4^+^ T cell count, a study conducted in Rwanda reported an association between HBV vaccine response in HIV-1-infected children and adolescents with CD4^+^ T cell counts higher than 350 cells/μL and a viral load below 40 RNA copies/mL [28]. An association between CD4^+^ T cell counts and response to HBV vaccine was also observed in adults with AIDS receiving an efavirenz-based ART regiment [29]. Moreover, a low HBV vaccine response rate in HIV-1 infected children, compared to controls, was associated with low frequencies of CD4^+^ T cells [30].

Regarding a gender effect, the likelihood of responding to the HBV vaccine was higher in adult HIV-1-infected women, compared to men, when these patients presented with CD4^+^ T cell counts >350 cells/μL [31]. In another study, the non-responder rate was lower in girls compared to boys among Malawian HIV-1 infected children; the rate of protected individuals and the levels of anti-HBs declined over time in children vaccinated at birth [19].

**Figure 1:**
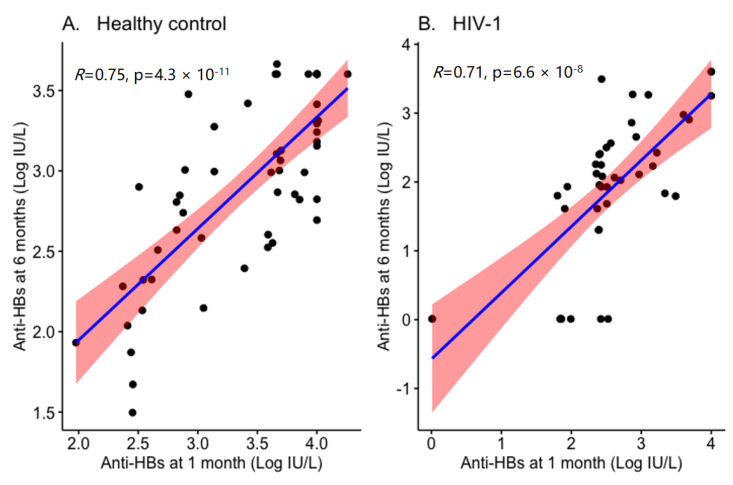
Correlation of anti-HBs titer at 1 and 6 months from completed HBV vaccination. Three doses of HBV vaccine were administered to children who were negative for HBsAg and previously unvaccinated, and periphery blood was collected at 1 month and 6 months after the last vaccination. Direct correlation of anti-HBs titers at 1 month and 6 months from the last vaccination dose in both healthy controls (**A**) and ART-treated HIV-1 infected (**B**) children. Anti-HBs: Hepatitis B surface antibody; IU/L: international units per liter (Modified from [8,33]).

Initial response to the vaccine was also predictive of sustained vaccine responses. Lopes and colleagues [32] reported that in HIV-1 infected individuals the persistence of protective anti-HBs antibody titers relates to the response levels at primary vaccination; in addition, in patients with a pronounced (>1000 IU/I) vaccine response at primary vaccination, the mean time loss of effective anti-HBs titer was 4.4 years [32]. Similarly, we have also found a direct correlation between anti-HBs antibody titer at 1 month and 6 months from the last dose of HBV vaccination in Ethiopian children (Figure 1). The immunological correlates presented by Lopes et al., (2013) remain somehow unclear, as a high CD4^+^ T cell count paradoxically was inversely correlated to a strong response at primary vaccination.

Regarding the role of ART initiation, the response rate to HBV vaccination in a study conducted in Tanzania was significantly lower in ART-naïve compared to ART-treated HIV-1 infected children with CD4^+^ T cell counts strongly predicting the levels of anti-HBs antibodies [34]. This latter result also supports a role for CD4^+^ T cells. The potential role of a CCR5 agonist-based ART regimen in improving immune responses to a double dose intramuscular (IM) HBV vaccination in HIV-1 infected adult patients was investigated [35]. At 1 month from the last vaccination, approximately 90% of the patients had responded to the vaccine and 81% maintained protective antibody levels at 1 year from vaccination [35]. Moreover, the CCR5 agonist-based ART regimen was associated with a wider vaccine response in patients younger than 50 years [35].

**Figure 2:**
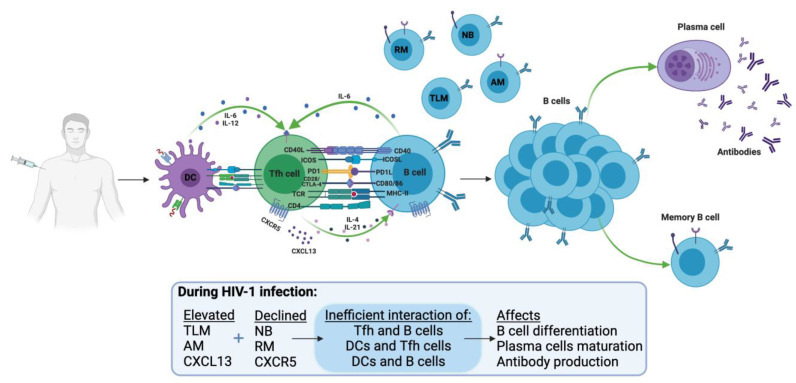
Immune responses to HBV vaccine. Antigen captured and processed by antigen-presenting cells (APCs); the processed antigen activates B cells and T cells. B-Tfh cells interact at the border of the GC for the generation of short-lived plasma cells and antibodies, and in the GC for the generation of long-lived plasma cells and development of memory B cells. In HIV-1 infection, inefficient interaction between B-Tfh cells was reported due to exhaustion of cells by direct and indirect effects of HIV-1. NB: naïve B cells, MB: memory B cells; AM: Activated memory B cells; TLB: Tissue-like memory B cells; Tfh: T follicular helper; IL: interleukin; GC: germinal center (Created with BioRender.com).

The read-out of a successful HBV vaccination is a vaccine-specific antibody response against HBsAg. Severe damage takes place in B cells, which are the cells devoted to antibody production, in HIV-1-infected individuals, both in children and adults [8,36]. This aspect of HIV-1 immunopathology may compromise the response to the HBV vaccine and also the maintenance of protective antibody titers to the HBV vaccine (Figure 2). The major abnormalities found in blood B cells during HIV-1 infection comprise a depletion of the general pool of memory B cells and an expansion of anergic non-typical B cell populations [37].

It is of interest that an expansion of an atypical memory CD19+CD10-CD27-CD21- subset of B cells, expressing high levels of the inhibitor FcRL5 receptor, can be found in HBV-infected patients [38]. HBV appears to have a direct role in compromising B cell function and induction of an atypical B cell phenotype. PD-1 blockade of atypical B cells isolated from chronically HBV-infected individuals could partially restore the biological properties of B cells including homing, cytokine, and antibody production [39]. In a study recently conducted in our laboratory, we showed that primary response to HBV vaccine, measured by antibody titers to HBs antigen, correlated with the frequency of resting and switched memory B cells in HIV-1 infected children [33]. Restoration of B cell function in HIV-1 infected individuals initiating ART during the chronic phase of infection may not be possible [40] and will likely require an early control of HIV-1 viremia resulting from early and prolonged adherence to ART, a strategy not always possible to implement in poor economic settings.

## 4. Booster or the Additional Dose of HBV Vaccine for HIV-1 Infected Individuals

Long-term protection and anamnestic response were reported after childhood HBV vaccination in immunocompetent individuals (Table 1). Protective anti-HBs titers were measured in only 37% of healthy participants (*n* = 300) attending the health center of Rafsanjan (Kerman province, southeast of Iran) 20 years from primary vaccination [41]; a single booster was administered to participants who had less than 10 IU/L of anti-HBs and the majority (>97%) responded to re-vaccination, suggesting the presence of an anamnestic response in previously vaccinated immunocompetent individuals [41]. The study shows that in immunocompetent individuals, loss of protective antibody does not necessarily imply the absence of immunity against HBV and contributes to the discussion on whether a booster dose of HBV vaccine should be administered to immunocompetent individuals [42]. Other studies also confirm that administration of booster dose(s) may not be necessary for these individuals and that childhood HB vaccination is sufficient to prevent HBV infection [22,43,44,45,46]. However, further studies should be conducted to prevent breakthrough infection of vaccine escape mutants in immunocompetent individuals living in the hyperendemic regions.

Post-vaccination screening for HBV vaccine-specific antibody levels in HIV-1 infected individuals followed by administration of a booster dose may be a sustainable strategy to prevent HBV transmission [52,53].The major challenge of accelerated vaccination, the common schedule in resource-limited settings, is the shorter longevity of induced antibodies compared to standard vaccination; this observation suggested that an accelerated vaccine schedule should be supplemented with an additional dose at 12 months from the last vaccination [54,55]. In this context, it should be taken into consideration that the waning of HBV antibodies is faster in HIV-1-infected compared to healthy individuals [8]; it is, therefore, likely that longevity of vaccine efficacy in this group can be preserved only by providing an additional dose at 1 year from the last vaccination point and possibly again at later time points.

Seven to 25 years from initial HBV vaccination at birth, HIV-1-infected adolescents and young adults who did not present with protective anti-HBs were provided a single IM dose of HBV vaccine; 50% responded, and the response rate was associated with CD4+ T cell counts [56]. An anamnestic response was measured in 82% of HIV-1 infected children who originally had anti-HBs antibodies below protective levels 3 years after revaccination [57] and in half of the HIV-1-infected children experiencing a gradual decline of anti-HBs at 8 weeks after vaccination [58]. These results show that a single HBV vaccine dose may reactivate a serological response in individuals presenting with insufficient levels of protective anti-HBs antibodies. In chronic kidney disease patients, non-responsiveness to recommended HBV vaccination, lower anti-HBs antibody titer, and rapid loss of protective antibodies were documented [59]. These patients who were in pre-dialysis and dialysis benefited from a booster dose to prevent HBV transmission since there is a higher potential to acquire the virus through contaminated dialysis equipment and surfaces [59,60,61]. Similarly, HIV-1 infected individuals had a suboptimal HB vaccine response and a higher chance of acquiring HBV with a serious outcome. Thus, a regular follow-up assessing maintenance of HBV vaccine responses should be implemented for HIV-1-infected individuals, and a booster dose should be given when titers fall below a protective threshold, to prevent HBV infection in this group of immunocompromised individuals.

## 5. Conclusions

In summary, a rapid waning of specific HBV vaccine antibodies is a major obstacle for HBV vaccination of HIV-1-infected individuals. Although the immunological mechanisms for loss of HBV vaccine antibodies during HIV-1 infection remain poorly characterized, emphasis should be given to impaired B cell immunology found in HIV-1-infected individuals, and chronic HBV infected patients. Searching for biomarkers of HBV vaccine response in HIV-1-infected individuals and the biological and immunological properties of B and T follicular helper (Tfh) cells, important immunological players in vaccine responses should be further evaluated. These future studies should include molecular signatures of B cell signaling and responses which were recently identified [62].

ART administration and adherence remain crucial to control viremia, maintain CD4^+^ T cell count, and restore immunological functions in HIV-1 infected individuals, including responses to vaccines. In addition to effective ART, vaccination schedules and vaccine doses should be revised to increase the response rate and longevity of HBV vaccine responses in HIV-1 infected individuals. Maintenance of HBV vaccine responses in HIV-1-infected individuals can be achieved only through regular serological monitoring and eventually provision of HBV vaccine booster doses. Priority should be given to these strategies even in less economically privileged settings to ensure the protection of HIV-1-infected children and adults against HBV.

## Figures and Tables

**Table 1:** Hepatitis B vaccine response in healthy individuals.

No	Schedule at Birth	Duration of Follow-Ups (Years)	Anamnestic Response and Timing	Response Rate for Non-Responder upon the 2nd Dose	Country	References
1	2, 4 & 6 months age	9 months to 16 years	Single-dose 10 μg. At 1 month: 95% for pre-booster detectable anti-HBs. 85% for undetectable pre-booster level.	3 doses; 92.3%	Egypt	[43]
2	0, 1, and 6 months	5–15 years	0–1–6 months, 10 μg. For < 10 mIU/mL *: 95.65% at 1 month. For > 10 mIU/mL *: 100% at 1 month.	NA	China	[47]
3	3 doses, at birth or 12 years	17.2 and 19.3 years after the last vaccination	Single-dose; at 1 month 88.8%.	Another dose, 100%	Italy	[48]
4	3 or more doses, at birth or young age		Single-dose; at 1 month 89.9%.	2 more doses; 96.2%	Italy	[49]
5	4 doses; First 2 years of life	Aged 14–15 years	Single-dose 10 µg. At 1 month. For < 6.2 mIU/mL *: 82.9%. For > 6.2–<10 mIU/mL *: 100%. For > 10 mIU/mL *: 98.6%.	NA	Germany	[50]
6	3 doses, at birth or during adolescence	18–20 years	Single-dose 10 μg. At 1 month: 97.7% for pre-booster detectable anti-HBs. 88.8% for undetectable pre-booster level.	2 more doses; 100%	Italy	[51]
7	3 doses; first year of life (2 days of birth, 1.5 and 9 months)	20 years	Single-dose 20 μg; 97.1% after 4 weeks.	NA	Iran	[41]
8	3–4 doses; all vaccinated during adulthood	20–30 years	Single-dose 20 μg. For < 10 mIU/mL *: 70% at day 7 and 100% at 1 month. For > 10 mIU/mL *: 100% at both day 7 and 1 month.	NA	Canada and Belgium	[44]

* Levels of anti-HBs antibody before booster dose/s.

## Data Availability

All data in this review were collected form published data and they are available in the original publications.
